# A pragmatic multi-center trial of goal-directed fluid management based on pulse pressure variation monitoring during high-risk surgery

**DOI:** 10.1186/s12871-017-0356-9

**Published:** 2017-05-30

**Authors:** Luiz Marcelo Sá Malbouisson, João Manoel Silva, Maria José Carvalho Carmona, Marcel Rezende Lopes, Murilo Santucci Assunção, Jorge Luís dos Santos Valiatti, Claudia Marques Simões, José Otavio Costa Auler

**Affiliations:** 1 Divisão de Anestesia, Hospital das Clínicas da Faculdade de Medicina da Universidade de São Paulo, Av. Enéas Carvalho de Aguiar, 255 2° andar, Cerqueira César, 05403-900 São Paulo, SP Brazil; 2 Hospital Padre Albino of Catanduva Medical School, Catanduva, SP Brazil; 3 Hospital São Paulo – São Paulo Federal University, São Paulo, SP Brazil

**Keywords:** Goal-directed fluid therapy, Hemodynamics, High-risk surgery, Pulse-pressure variation, Postoperative complications

## Abstract

**Background:**

Intraoperative fluid therapy guided by mechanical ventilation-induced pulse-pressure variation (PPV) may improve outcomes after major surgery. We tested this hypothesis in a multi-center study.

**Methods:**

The patients were included in two periods: a first control period (control group; *n* = 147) in which intraoperative fluids were given according to clinical judgment. After a training period, intraoperative fluid management was titrated to maintain PPV < 10% in 109 surgical patients (PPV group). We performed 1:1 propensity score matching to ensure the groups were comparable with regard to age, weight, duration of surgery, and type of operation. The primary endpoint was postoperative hospital length of stay.

**Results:**

After matching, 84 patients remained in each group. Baseline characteristics, surgical procedure duration and physiological parameters evaluated at the start of surgery were similar between the groups. The volume of crystalloids (4500 mL [3200-6500 mL] versus 5000 mL [3750-8862 mL]; *P* = 0.01), the number of blood units infused during the surgery (1.7 U [0.9-2.0 U] versus 2.0 U [1.7-2.6 U]; *P* = 0.01), the fraction of patients transfused (13.1% versus 32.1%; *P* = 0.003) and the number of patients receiving mechanical ventilation at 24 h (3.2% versus 9.7%; *P* = 0.027) were smaller postoperatively in PPV group. Intraoperative PPV-based improved the composite outcome of postoperative complications OR 0.59 [95% CI 0.35-0.99] and reduced the postoperative hospital length of stay (8 days [6-14 days] versus 11 days [7-18 days]; *P* = 0.01).

**Conclusions:**

In high-risk surgeries, PPV-directed volume loading improved postoperative outcomes and decreased the postoperative hospital length of stay.

**Trial Registration:**

ClinicalTrials.gov Identifier; retrospectively registered- NCT03128190

## Background

Major surgeries in high-risk patients are associated with the development of postoperative complications, with mortality rates ranging from 15 to 30% [1–3]. Intraoperative hemodynamic strategies aiming to maintain adequate oxygen delivery have been shown to reduce morbidity, mortality, and postoperative length of hospital stay in several publications [4–7].

Despite the evidence favoring intraoperative hemodynamic optimization in high-risk patients, [8] the role of each intervention in increasing oxygen delivery remains unclear. This is particularly true of fluid loading. Volume management in the perioperative period plays a pivotal role in resuscitation and, hence, morbidity and mortality in surgical patients [2]. Excessive fluid administration may aggravate pulmonary dysfunction, prolong the need for mechanical ventilation, extend the hospital length of stay and increase postoperative mortality [9].

Accurate prediction of fluid responsiveness may identify patients who would benefit from volume expansion, and prevent unnecessary fluid loading [10, 11]. Previous studies have demonstrated that pulse pressure variation (PPV) is an accurate predictor of fluid responsiveness during mechanical ventilation [12, 13]. Patients who have reached the plateau of the Frank-Starling relationship can be identified as patients in whom PPV is lower than 13% [14]. The intraoperative goal of maximizing stroke volume by volume loading can therefore be achieved simply by maintaining a PPV below 13% [13, 15]. In this study, we investigated whether PPV-guided intraoperative fluid loading in high-risk surgical patients improves postoperative outcomes.

## Methods

This open label, multicenter, before-and-after trial study was carried out in three hospitals, Hospital das Clínicas of São Paulo University Medical School, Hospital São Paulo of São Paulo Federal University and Hospital Padre Albino. An independent Data Monitoring Committee reviewed unblinded data for patient safety, and after the pilot trial [13], this committee recommended that a before-and-after study would be more adequate to avoid clinical evidence possibility of inadequate treatment in control group. The protocol was immediately amended in accordance with that recommendation, and participants were subsequently assigned in before and after periods.

Subsequently obtaining IRB approval (Ethical Committee N° 0616/06 HCFMUSP) and written informed consent, high-risk patients undergoing open major surgery under general anesthesia and who required ICU admission postoperatively were enrolled from June 2007 to April 2008 (control period), followed by a phase-out period (April 2008 to July 2008) and an intervention period (July 2008 to June 2010).

High-risk surgical patients were defined as those 60 years of age or older referred to postoperative ICU care due to the presence of at least one clinical comorbidity such as coronary artery disease, chronic obstructive pulmonary disease, cerebrovascular disease, poor nutritional status, a predicted intraoperative period greater than 6 h or predicted acute massive blood loss. All of these criteria have been used in previous studies, [2, 16] and they were adopted for this study. Patients with severe aortic regurgitation, cardiac arrhythmias, congestive heart failure, patients undergoing renal replacement therapy, those undergoing palliative surgery were excluded from the study.

Patients were enrolled into a standard fluid resuscitation period (Control group) or a goal-directed fluid-management group based on pulse pressure variation during the intervention period (Intervention group). Following the control period, the three-month phase-out period included education and preparation of all anesthesiology staff and logistic arrangements for the evaluation of fluid responsiveness using automated PPV measurements. Finally, the intervention period was replicated in the same season of the year as the control period.

### Anesthesia care and fluid management

Intraoperative monitoring standards for high-risk patients in all institutions included electrocardiography, invasive arterial blood pressure catheters, pulse oximetry, temperature monitoring, and measurement of inspiratory and expiratory gas concentrations. Additional intraoperative monitoring such as central venous catheterization was indicated on an individual basis by the attending anesthesiologist. In the interventional phase, however, intraoperative fluid adjustment was strictly directed by online PPV assessment.

In the control period, patients were given intravenous fluids at the discretion of the anesthesiologist based on institutional protocol using 250 ml of crystalloids or 100 ml of colloids based on central venous pressure (CVP) and mean arterial pressure (MAP) measurements. The aim was to keep the CVP ≥ 8 mmHg and MAP ≥ 65 mmHg. Fluid boluses were administered up to a total of 1000 ml, if patients did not attain a MAP of >65 mmHg, a vasopressor drug was administered. During this initial phase, the anesthesiologists were blind to the enrollment of the patient in the study. In the interventional phase of the study, fluids boluses of colloids were given to maintain continuously measured PPV at 10% or less [13]. Some studies [17, 18] identified different PPV cutoffs values and a higher cutoff value may result in a substantial number of false-negative results, meaning that necessary volume loading is withheld from patients who would benefit from fluid administration. When dealing with a population of high-risk surgical patients, fluid loading is more important to correct hemodynamic problem than in a normal population. For this reason, a cutoff point based on a PPV of 10% is justified.

In order to evaluate PPV during surgery, an arterial line was connected to a monitor (DX 2020, Dixtal, São Paulo, SP, Brasil) specifically developed to detect respiratory variations in the arterial pressure curve, allowing for the automatic calculation of beat to beat pulse pressure, as previously described [13, 15]. PPV was calculated using the following formula:

$$ \mathrm{P}\mathrm{P}\mathrm{V}=100\times \left(\mathrm{PPmax}\hbox{-} \mathrm{PP} \min \right)/\left[\left(\mathrm{PPmax}+\mathrm{PPmin}\right)/2\right] $$

The mean value of PPV was automatically calculated over three consecutive floating periods of eight respiratory cycles and the median value of this triple determination was displayed on the multiparameter monitor and updated after each new respiratory cycle [13]. The shape of the arterial curve was checked visually for damping throughout the study period. The respective hemodynamic protocols in both groups were continued until the end of surgery.

In the interventional period, the mechanical ventilator settings were adjusted using the following parameters: a) a tidal volume of 8 mL.kg^-1^ (ideal body weight) in volume control mode; b) an inspiratory time of 33% of the respiratory cycle; c) 5 cmH_2_O positive end expiratory pressure and d) respiratory rate adjusted to maintain an end-tidal capnometry of 35 mmHg to enable the measurement of PPV.

During the postoperative period, critical care and ward teams not involved in the intraoperative management or in data collection managed the patients. These individuals were not informed of patient allocation groups or study period.

### Data Collection and Monitoring

During the study, an investigator not participating in patient care collected all study data prospectively up until hospital discharge or patient death. Age, weight, height, sex, comorbidities such as cirrhosis, chronic obstructive pulmonary disease, hypertension, peripheral vascular disease, coronary artery disease, other cardiac disease, diabetes mellitus and cerebrovascular disease were recorded preoperatively as well as standard routine biochemical blood tests were performed. Mechanical ventilation settings, PPV values at 30 min’ intervals, the use of vasopressors and inotropes and the duration of surgery were recorded during intraoperative. Heart rate (HR), mean arterial pressure (MAP), peripheral capillary oxygen saturation (SpO_2_), hemoglobin concentration and esophageal temperature were registered at the beginning and at the end of the surgical procedure. When available, central venous pressure (CVP) was recorded at the end of the surgery. The total volumes of crystalloids, colloids and blood products, percentage of patients receiving red blood cell transfusion, and use of vasopressors and inotropic drugs were recorded.

After ICU admission and 24 h later, the following parameters were collected: HR, MAP, SpO_2_, CVP and arterial lactate concentration. In the ICU, the total volumes of infused crystalloids, colloids and hemocomponents were recorded as well as the percentage of patients receiving red blood cell pack (RBCP) unit transfusion. Postoperative complications were assessed daily until patient discharge according to previously published criteria [13]: 1) vasopressor need was named circulatory shock defined by the need for continuous norepinephrine infusion after adequate fluid adjustment; 2) major ICU infections (lung, abdominal, urinary tract, line-related sepsis or wound infections); 3) respiratory dysfunction, defined as recently as recent partial pressure of oxygen in arterial blood/fraction of inspired oxygen (FiO_2_) of <200 without prior patient history; 4) need for reoperation; 5) need for mechanical ventilation; 6) hematologic dysfunction, defined as a platelet count of < 100,000/μL or prothrombin activity of <50%; 7) bleeding events that needed transfusion of platelets or coagulation factors; 8) renal dysfunction, defined as a urine output of <500 mL/day, a serum creatinine level of >1.9 mg/dL, or dialysis for acute renal failure; and/or; 9) hepatic dysfunction, defined as a serum bilirubin level of >1.9 mg/dL. Postoperative length of stay and mortality were also recorded.

Upon completion of data collection from each patient, an independent Data Safety and Monitoring Board (DSMB) member conducted data quality monitoring by comparing the collected study information to the information contained in institutional medical records.

### Data analysis

The primary outcome of this study was postoperative length of hospital stay. The secondary outcomes included the volume of infused fluids, RBCP transfusion, incidence of organ dysfunction, incidence of postoperative complications and a composite outcome encompassing postoperative complications and hospital mortality rate. According to the literature [19] and by using the minimal clinically significant difference between groups, eighty-one patients were required in each group to find a reduction of 2 days (from 14 ± 5 days in the Control Group to 12 ± 4 days in the Intervention Group), with a Type-I error of 0.05 (one-sided) and a power of 0.8.

Normal data distribution was tested using the Kolmogorov-Smirnov test. Continuous data were compared between groups using Student’s *t*-tests or Wilcoxon signed-rank test. Repeated measurement variables were studied over time using a generalized estimating equation (GEE) model with the within factor being the time point of the study and the grouping factor being the study group. If main effects or interactions were significant, a pairwise *post-hoc* analysis using the Šidák test was performed. Binominal data were compared using chi-squared analysis and Fisher’s exact test. The Mantel–Haenszel hazard ratio and log-rank test were used to compare the postoperative length of hospital stay between the groups. The composite outcome was reported as an odds ratio (OR), representing the OR of the occurrence of at least one major complication over the odds of no occurrence of complications.

#### Controlling for confounding variables

Since data from the intervention and control groups were collected in two distinct periods in a non-randomized fashion, differences in patients’ baseline characteristics could have led to biased estimates of treatment effects. In order to balance the baseline characteristics and reduce bias, we matched patients from the intervention and control groups using a propensity score, defined as the conditional probability of being treated. First, a logistic regression model was created using the group variable as the dependent variable. Other potential confounding risk factors for morbidity and length of stay considered in the analyses included age, weight, time of surgery, and type of operation were entered as predictors, and the width of the matching tolerance caliper was set at 0.05 of the logit. Those variables were selected on matching by using a back-door criterion, which detected the presence of confounding variables. Then, a match for each intervention group patient was selected from the control group based on the closest logit. This model was constructed based on a sample of patients matched by propensity score 1:1 without replacement or repetition. The matching procedure was performed before the analysis of the study outcomes.

Differences were considered significant at *p* <0.05. The results are expressed as mean ± standard deviation, percentage, OR ± 95% confidence interval (CI) or as defined otherwise. All analyses were conducted using IBM-SPSS software (Version 21, IBM Corp. Armonk, NY, USA).

## Results

### Group allocation and matching

**Fig. 1:**
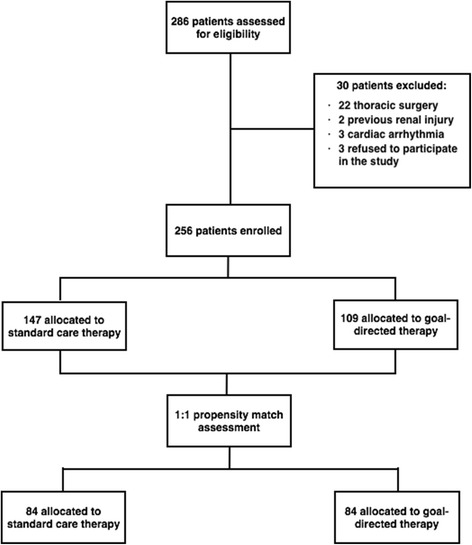
Patient flow throughout the study

During the study period, 256 surgical patients were enrolled (147 during the control period and 109 during the intervention period) (Fig. 1). The baseline characteristics of the patients are presented in Table 1. In the original cohort, it was observed that the incidence of non-dialytic renal failure, the white cell count, MAP and duration of surgery were significantly greater than in the PPV group, while the incidence of cirrhosis, tidal volume and initial intraoperative hematocrit were greater in the intervention group. The frequency of surgical procedures was also unbalanced between the groups (*P* = 0.04). After propensity score matching, 84 patients remained in each group and all the baseline characteristics regarding comorbidities (Table 1) and frequency of surgical procedures (Table 2) were well balanced.

**Table 1:** Baseline characteristics

	Original Cohort		Matched Cohort			
	Control (*n* = 147)	Intervention (*n* = 109)	*P*	Control (*n* = 84)	Intervention (*n* = 84)	*P*
Male, n (%)	91 (61.9%)	73 (67%)	0.40	53 (63.1%)	54 (64.3%)	0.87
Age (y), mean ± SD	71.1 ± 7.4	69.5 ± 7.0	0.07	69.5 ± 7.2	70.5 ± 7.0	0.39
Weight (kg), mean ± SD	66.1 ± 14.2	66.3 ± 13.6	0.87	65.9 ± 13.5	65.7 ± 13.9	0.92
ASA Score			0.16			0.76
P 2 score, n (%)	74 (51.3%)	63 (57.8%)		45 (53.6%)	46 (54.8%)	
P 3 score, n (%)	69 (46.9%)	46 (42.2%)		38 (45.2%)	38 (45.2%)	
P 4 score, n (%)	4 (2.7%)	-		1 (1.2%)	-	
Chronic diseases						
Non-dialytic renal failure, n (%)	32 (21.9%)	14 (12.8%)	0.03	16 (19%)	9 (10.7%)	0.13
Cirrhosis, n (%)	1 (0.7%)	5 (4.6%)	0.04	1 (1.2%)	3 (3.6%)	0.31
Chronic obstructive pulmonary disease, n (%)	21 (14.3%)	17 (15.6%)	0.77	13 (15.5%)	11 (13.1%)	0.66
Hypertension, n (%)	112 (76.2%)	88 (71.6%)	0.40	64 (76.2%)	65 (77.4%)	0.85
Coronary artery disease, n (%)	46 (31.3%)	27 (24.8%)	0.25	22 (26.2%)	24 (28.6%)	0.73
Other cardiac disease, n (%)	9 (6.1%)	5 (4.6%)	0.59	4 (4.7%)	5 (5.9%)	0.73
Diabetes mellitus, n (%)	38 (25.8%)	28 (25.7%)	0.89	26 (30.9%)	22 (26.2%)	0.61
Preoperative biological tests						
Blood urea nitrogen (mg/dL), mean ± SD	48.3 ± 24.7	43.2 ± 22.9	0.11	47.1 ± 22.3	42.6 ± 18.0	0.17
Creatinine (mg/dL), mean ± SD	1.3 ± 1.2	1.1 ± 0.7	0.25	1.2 ± 0.5	1.1 ± 0.7	0.62
Plasmatic sodium (mEq/L), mean ± SD	139 ± 4	138 ± 14	0.26	139 ± 4	139 ± 4	0.60
Plasmatic potassium (mEq/L), mean ± SD	4.4 ± 0.6	4.2 ± 0.4	0.07	4.3 ± 0.6	4.2 ± 0.5	0.76
Glucose (mg/dL), mean ± SD	110 ± 37	114 ± 39	0.58	120 ± 53	106 ± 31	0.17
Hemoglobin (mg/dL), mean ± SD	12.4 ± 1.9	12.4 ± 1.9	0.99	12.3 ± 1.9	12.2 ± 1.9	0.69
Hematocrit (%), mean ± SD	38.2 ± 5.5	37.6 ± 5.2	0.41	38.0 ± 5.8	37.2 ± 5.2	0.38
Platelets (count/μL), mean ± SD	248 ± 112	251 ± 04	0.85	250 ± 112	249 ± 104	0.93
Leukocytes (count/mL), mean ± SD	8002 ± 3355	6814 ± 2375	0.01	7805 ± 2910	6646 ± 2322	0.06
INR, mean ± SD	1.0 ± 0.2	1.0 ± 0.2	0.95	1.0 ± 0.2	1.1 ± 0.2	0.23

Legends: *ASA* American Association of Anesthesiologists, *INR* international normalized ratio

**Table 2:** Intraoperative data on control and intervention groups

	Original Cohort			Matched Cohort		
	Control (*n* = 147)	Intervention (*n* = 109)	*P*	Control (*n* = 84)	Intervention (*n* = 84)	*P*
Type of surgery			0.04			0.48
Gastrointestinal, n (%)	47 (32%)	59 (53.2%)		29 (34.3%)	47 (56%)	
Gynecological, n (%)	7 (4.8%)	3 (2.3%)		1 (1.2%)	3 (3.6%)	
Vascular, n (%)	65 (44.2%)	26 (23.9%)		39 (46.4%)	21 (25%)	
Urological, n (%)	15 (10.2%)	21 (19.3%)		10 (11.9%)	13 (15.5%)	
Combined interventions, n (%)	13 (8.8%)	1 (0.9%)		5 (6%)	-	
Respiratory settings						
PEEP (mmHg), mean ± SD	5.3 ± 1.5	5.5 ± 1 .1	0.47	5.2 ± 1.8	5.4 ± 1.1	0.31
Tidal volume (mL/kg), mean ± SD	7.6 ± 1.4	8.1 ± 1.2	0.003	7.7 ± 1.2	8.2 ± 1.2	0.05
RR (breaths/min), mean ± SD	11.5 ± 1.2	11.5 ± 1.2	0.94	11.5 ± 1.3	11.5 ± 1.2	0.92
FiO_2_ (%), mean ± SD	49 ± 8	49 ± 7	0.52	50 ± 7	48 ± 6	0.04
Physiologic status at start of surgery						
HR (BPM), mean ± SD	76 ± 13	71 ± 15	0.28	71 ± 12	71 ± 15	0.86
MAP (mmHg), mean ± SD	97 ± 15	88 ± 17	0.03	100 ± 11	91 ± 17	0.06
SpO_2_ (%), mean ± SD	95 ± 7	97 ± 2	0.10	95 ± 10	97 ± 2	0.11
Hematocrit (%), mean ± SD	29 ± 6	34 ± 7	0.03	30 ± 5	32 ± 7	0.24
Physiologic status at end of surgery						
HR (BPM), mean ± SD	74 ± 14	73 ± 14	0.52	74 ± 13	72 ± 13	0.45
MAP (mmHg), mean ± SD	83 ± 13	79 ± 16	0.11	82 ± 14	80 ± 16	0.65
CVP (mmHg), mean ± SD	12 ± 4	11 ± 7	0.68	12 ± 3	11 ± 8	0.93
SpO_2_ (%), mean ± SD	98 ± 1	98 ± 1	0.42	98 ± 1	98 ± 1	0.95
Hematocrit (%), mean ± SD	34 ± 6	33 ± 6	0.65	34 ± 6	33 ± 5	0.40
Fluid administered intraoperatively						
Crystalloids (mL), median (IQ 25–75)	4500 (3000–7000)	5200 (3500–6850)	0.24	5000 (3750–8862)	4500 (3200-6500)	0.01
Colloids (mL), median (IQ 25–75)	1000 (500-1000)	900 (500-1000)	0.46	1000 (500-1000)	900 (500-1000)	0.33
Number of patients transfused, n (%)	64 (43.5%)	51 (46.8%)	0.6	44 (52.4%)	39 (46.4%)	0.44
RBCP units/patient, median (IQ 25–75)	1.7 (0.9–2.5)	1.7 (0.9–2.0)	0.08	2.0 (1.7–2.6)	1.7 (0.9–2.0)	0.01
Intraoperative noradrenaline, *n* (%)	10 (6.8%)	18 (16.5%)	0.01	5 (3%)	9 (5.4%)	0.4
Intraoperative dobutamine, *n* (%)	2 (1.4%)	7 (6.4%)	0.04	1 (0.6%)	5 (3%)	0.21
Surgery length (min), mean ± SD	324 ± 147	410 ± 165	0.001	349 ± 141	355 ± 130	0.39

Legends: *PEEP* positive end-expiratory pressure, *RR* respiratory rate, *FiO*_*2*_ Inspired oxygen fraction, *HR* heart rate, *MAP* mean arterial pressure, *CVP* central venous pressure, *SpO*_*2*_oxygen saturation measured by pulse oximetry, *RBCP* red blood cell pack

The overall mean age was 70 ± 7.1 years old, and 64% of the matched population were men, without differences between groups. In the matched cohort, 53.6% of control group and 54.8% of intervention group were classified as P2 patients according to the American Society of Anesthesiology (ASA) classification, while 45.2% in both groups were classified as P3 (*P* = 0.76). Two or more preoperative comorbidities were present in 54.3% and 45.7% of the control and intervention groups, respectively (*P* = 0.49).

### Intraoperative physiological status, ventilator parameters and hemodynamic management in the propensity matched cohort

The duration of surgery, physiological parameters evaluated at the start and end of the surgery and ventilator settings used were similar in both groups (Table 2). Intraoperative FiO_2_ was slightly higher in the control group (50 ± 7% versus 48 ± 6%, P = 0.04) but not clinically relevant. At the end of surgery, central venous pressure was 12 ± 3 mmHg in the control group and 11 ± 8 mmHg in the intervention group (*P* = 0.93). The mean PPV was 7.4% in the intervention group throughout the study, and remained below 10% in both the original and matched cohorts. In the intervention group, the median volume of crystalloids infused was significantly lower than that in the control group (*P* = 0.01), as shown in Table 2. Conversely, both groups received a similar volume of colloids (*P* = 0.33). The fraction of patients receiving RBCP transfusion was similar in both groups (*P* = 0.44), but a greater number of RBCP units were transfused *per* patient in the control group (*P* = 0.01). The need for vasopressor and inotropic drug infusion and the hematocrit concentration measured at the end of surgery were similar in both groups.

### Postoperative outcomes in the propensity matched cohort

Upon ICU admission and 24 h later, the MAP and HR were similar in both groups (Table 3). However, the median value of CVP was lower in the intervention group after 24 h in the ICU (*P* = 0.002). The volume of crystalloid solution infused (*P* = 0.001) and number of patients receiving RBCP units (*P* = 0.003) on the first postoperative day were significantly greater in the control group in comparison to the intervention group, as shown in Table 3. After 24 h in the ICU, 9.7% of patients in the control group still required mechanical ventilation compared with 3.2% of the intervention group (*P* = 0.027).

**Table 3:** Postoperative outcomes

	Original Cohort			Matched Cohort		
	Control (*n* = 147)	Intervention (*n* = 109)	*P*	Control (*n* = 84)	Intervention (*n* = 84)	*P*
At the time of ICU admission						
HR (BPM), mean ± SD	77 ± 16	79 ± 14	0.51	79 ± 16	77 ± 14	0.40
MAP (mmHg), mean ± SD	93 ± 20	89 ± 20	0.25	89 ± 22	90 ± 20	0.77
Lactate (mmol/l), median (IQ 25–75)	2.1 (1.5 - 3.5)	2.6 (1.8 - 3.7)	0.48	2.6 (1.8-4.3)	2.5 (1.5-3.2)	0.55
CVP (mmHg) median (IQ 25–75)	10 (6.2 - 13.0)	9 (5 – 10)	0.04	10 (6.0 – 13)	9 (5-10)	0.13
At 24 h after ICU admission						
HR (BPM), mean ± SD	80.3 ± 16.4	83.5 ± 17.3	0.15	82.3 ± 15.9	81.9 ± 16.3	0.87
MAP (mmHg), mean ± SD	85.4 ± 15.1	81.7 ± 13.8	0.08	86.2 ± 15.4	82. 7 ± 14.4	0.21
Lactate (mmol/l), median (IQ 25–75)	2.4 (1.8 - 3.2)	1.8 (0.7 - 2.7)	0.07	2.3 (1.6-2.9)	2.3 (1.1-2.7)	0.41
CVP (mmHg), median (IQ 25–75)	11 (8.0 – 15)	8 (6.0 - 10.2)	0.00	12 (8.5-15)	8 (6.25-11)	0.002
Fluid administered in first 24 h						
Crystalloids (mL), median (IQ 25–75)	3630 (2685 - 4822)	3500 (2412- 4450)	0.13	4343 (3154- 5620)	3515 (2400 - 4270)	0.00
Colloids (mL), median (IQ 25–75%)	500 (500 - 1000)	500 (500 - 500)	0.09	500 (500 - 1000)	500 (500 - 500)	0.23
Number of patients transfused, n (%)	41 (27.9%)	17 (15.6%)	0.02	27 (32.1%)	11 (13.1%)	0.003
RBCP units/patient, median (IQ 25–75)	1.7 (0.8 – 2.0)	1.0 (0.8 – 2.6)	0.71	0.8 (0.8 – 2.4)	1.0 (0.8 - 3.5)	0.44
ICU LOS (d), median (IQ 25–75)	2 (1-4)	2 (1-4)	0.59	3 (1 – 4)	2 (1 – 4)	0.29
Postoperative LOS (d), median (IQ 25–75)	9 (6-15)	8 (6.25-14)	0.83	11 (7 – 18)	8 (6 – 14)	0.01
Hospital LOS (d), median (IQ 25–75)	17 (11-26)	16 (9-25)	0.30	22 (13 - 30.75)	15 (9 - 23.75)	0.01
Hospital mortality rate	26 (17.7%)	16 (14.8%)	0.54	16 (19.0%)	11 (13.3%)	0.31

Legends: *HR* heart rate, *MAP* mean arterial pressure, *CVP* central venous pressure, *SpO*_*2*_ peripheral oxygen saturation, *RBCP* red blood cell pack, *ICU* intensive care unit, *LOS* Length of Stay

**Fig. 2:**
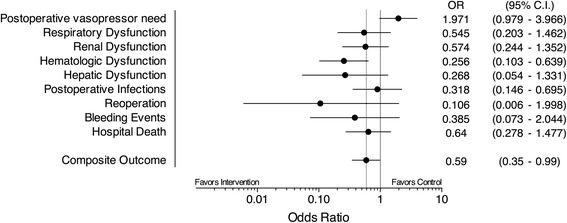
Odds ratios and 95% confidence intervals for clinical outcomes

The postoperative complication odds ratios are shown in Fig. 2. Despite a trend for an increased incidence of cardiovascular dysfunction manifest in the need for continuous infusion of vasopressors during the first 24 h postoperatively in patients receiving fluids directed by PPV monitoring (OR: 1.97; 95% CI: 0.97 – 3.96), postoperative arterial lactate concentrations, an indicator of tissue hypoperfusion, were similar between the Control and Intervention Groups upon ICU admission (*P* = 0.55) and after 24 h (*P* = 0.41), as shown in Table 3. The incidence hematological dysfunction was lower in the intervention group (OR 0.26; CI 95% 0.1 – 0.64). A trend towards a lower incidence of respiratory (OR: 0.54; 95% CI: 0.2–1.46), renal (OR: 0.57; 95% CI: 0.24–1.35), and hepatic (OR: 0.27; 95% CI: 0.05–1.33) organ failures in the Intervention Group was also evident. Combining all postoperative complications and hospital deaths into a composite postoperative complication outcome revealed that PPV-guided intraoperative fluid loading reduced the morbidity of high-risk patients undergoing major surgical interventions (OR: 0.59; 95% CI: 0.35–0.99).

**Fig. 3:**
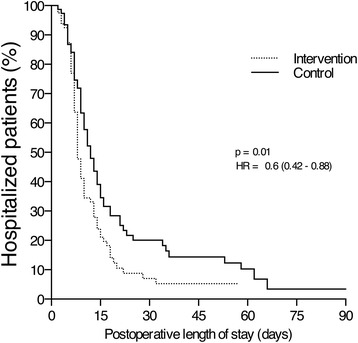
Postoperative length of stay curves of the propensity matched control group (*solid line*) and of the propensity matched intervention group (*dotted line*) plotted using the Kaplan-Meyer method

Although no differences were apparent in the median length of ICU stay between the Control and Intervention Groups (3 days (1 – 4) vs. 2 days (1 – 4); *P* = 0.29), the median postoperative and hospital length of stay were significantly shorter in the intervention group, as shown in Table 3. The hazard ratio for the postoperative length of hospital stay was 0.6 (95% CI: 0.42 - 0.88) in the patients receiving intraoperative fluid loading guided by PPV (Fig. 3). Mortality rates were not statistically different in the control (19%) and in intervention (13.3%) groups.

## Discussion

In this study, we observed that PPV-guided intraoperative fluid loading was associated with a decrease in postoperative complications and in hospital length of stay. In addition, we observed a reduction in the amount of fluids and RBCP units infused during surgery and in the first 24 h postoperatively.

Goal-directed hemodynamic therapy (GDHT) aiming to optimize intraoperative oxygen delivery has been shown to reduce the incidence of postoperative organ dysfunction and improve short- and long-term outcomes in high-risk surgical patients in several studies [2, 7, 20–23]. In a meta-analysis of 31 studies published in 2012 [24], the authors reported that GDHT reduced the number of patients with postoperative complications and the length of hospital stay by 1.16 days. Pearse et al. [25] included their data in an updated meta-analysis, and showed that GDHT was associated with a reduction in complication rates, postoperative infections, and duration of hospital stay.

Despite growing evidence pointing to the role of such hemodynamic optimization strategies to reduce the incidence of postoperative morbidity in high-risk surgical patients, the best intraoperative fluid management strategy remains controversial [11, 26]. During surgery, hypovolemia occurs as a consequence of extended preoperative fasting, bleeding and loss of fluids to the interstitium due to the systemic inflammatory response [27]. Moreover, general anesthesia blunts compensatory autonomic responses to an oxygen delivery/consumption mismatch. Standard hemodynamic monitoring such as central venous pressure monitoring and surrogates of tissue perfusion adequacy such as lactate and central venous saturation have a low power to discriminate patients whose cardiac output will increase in response to volume loading [10, 28]. Furthermore, only monitoring surrogates of cardiovascular performance without using a treatment algorithm has proven to be ineffective in facilitating hemodynamic stabilization or affecting outcome [29].

Our results showed that goal-directed fluid management based on PPV monitoring during high-risk surgery reduced the total amount of fluids given intraoperatively and the blood transfusion volume while arterial lactate concentrations measured immediately after ICU admission and 24 h later were similar in both groups. These findings point to a thoroughly different scene from previous GDHT studies in which the control groups presented intraoperative hypoperfusion and consequent worse outcomes [4, 13, 30]. The comprehension of intraoperative hypoperfusion as the underlying mechanism of postoperative organ failure and the consequent need to maintain adequate intraoperative oxygen transport raises a new question in intraoperative fluid management, it means, how much fluid is enough to optimize tissue perfusion and prevent postoperative organ dysfunction while avoiding unwanted consequences. In a recent study [9], it was observed that excessive intraoperative fluid infusion resulted in greater organ dysfunction and infection rates in high-risk surgical patients. Other authors have also reported the same association between excessive fluid infusion and increased mortality, morbidity and length of hospital stay in this population [2, 31]. However, in contrast to previous studies comparing restrictive versus liberal intraoperative fluid therapy, [32, 33] by using PPV to guide fluid resuscitation, we were able to maximize cardiac performance and oxygen delivery while avoiding complications associated with fluid overload.

Apart, in a pilot study using a before-and-after methodology of PPV in patients undergoing cardiac surgery, Suzuki *et* [34] found a small increase in the amount of fluids given by using PPV method, but there was very little evidence of any difference in physiologic or clinical outcomes.

Our study has some important limitations that require careful interpretation. First of all, it was not a blinded randomized trial. Unfortunately, it is practically impossible for it to be blinded, because the evaluation of intraoperative hemodynamic interventions using online monitoring devices may introduce observer bias into randomized controlled trials. Therefore, we designed a prospective before-and-after study with an “anesthesiologist-blinded” in control group to evaluate the impact of intraoperative PPV-guided fluid management on postoperative outcomes instead of a standard randomized controlled trial. It would be practically impossible to blind the study intervention, since the arterial pressure curve must be on the monitor to enable anesthesiologists to assess the quality of pressure monitoring. Hence, a mere glance at the arterial pressure waveform would give information on fluid responsiveness and potentially alter the way that fluid therapy decisions were made in the Control Group [35]. Moreover, the simple fact of knowing that patients were included in a study would modify the way caregivers guide therapy of the control group (Hawthorne effect), therefore distorting the true impact of intraoperative PPV-guided intervention. Conversely, we enrolled patients at the same time of the year, ruling out a seasonal effect. Taking into account these issue, a before-and-after evaluation is the most appropriate design to investigate intraoperative PPV-guided fluid administration.

Second, nonetheless, the absence of randomization introduces other problems such as the possibility of selection bias from data collected in two distinct periods and imbalances between the groups leading to biased estimates of treatment effects. In order to balance the baseline characteristics and reduce bias, we matched patients from the intervention and control groups using 1:1 propensity score matching without repetition, even though there is the risk of unmeasured confounding in the propensity score matched analysis. While the sample included in this study was relatively small and the caliper used to match the groups was 0.05 of the logit of the logistic regression model, the groups were well balanced. Nevertheless, propensity scoring is limited by calculating the probability of receiving the treatment based on observed and measured variables. Hence, although superior to traditional covariance analysis methods used to decrease bias in observational data, it does not account for unobserved covariates that may be relevant but not present in the dataset but assumes that the probability is based solely on the covariate score. Lastly, since the project design was older, some definitions about complication/outcomes did not follow current recommendations in perioperative outcome measures [36].

## Conclusion

In summary, these findings suggest that the intraoperative use of PPV minimally invasive monitoring and a fluid optimization protocol seems to decrease post-operative complications and hospital length of stay.

## Abbreviations

ASA: American Society of Anesthesiologists
CI: Confident interval
CVP: Central venous pressure
DSMB: Data safety and monitoring board
GDHT: Goal-directed hemodynamic therapy.
GEE: Generalized estimating equation
HR: Heart rate
ICU: Intensive care unit
IRB: Institutional ethical board
LOS: Length of stay
MAP: Mean arterial pressure
OR: Odd ratios
PaO_2_/FiO_2_: Ratio partial pressure of oxygen/fraction of inspired oxygen
PPmax: Pulse pressure maximum
PPmin: Pulse pressure minimum
PPV: Pulse-pressure variation
RBCP: Red blood cell pack units
SpO2: Pulse oximeter
